# Separation of sedimentary phytoliths from other biogenic silica particles for triple oxygen isotope analysis

**DOI:** 10.1016/j.mex.2025.103373

**Published:** 2025-05-17

**Authors:** Charlotte Mention, Julie C. Aleman, Jean-Charles Mazur, Yannick Garcin, Christelle Hély, Anne Alexandre

**Affiliations:** a Ecole Pratique des Hautes Etudes (EPHE), PSL University, Paris, France; b CNRS, Aix Marseille Univ, IRD, INRA, CEREGE, Aix-en-Provence, France; c ISEM, Univ Montpellier, CNRS, IRD, EPHE, Montpellier, France

**Keywords:** Phytoliths, Diatom frustules, Sponge spicules, Oxygen isotope, Filtration, Concentration, Phytoliths concentration by separating from others silica particles by filtration

## Abstract

Phytoliths are amorphous silica particles that precipitate within and between plant cells, and their fossilized morphological assemblages are widely used to reconstruct paleo-vegetation. The triple oxygen isotope composition of phytoliths, expressed by the ^17^O-excess, is a promising proxy to reconstruct atmospheric relative humidity (RH). However, fossil phytoliths in lake or peat sediments often coexist with diatom frustules and sponge spicules, whose oxygen isotope signatures contribute to the average isotopic composition of biogenic silica, biasing the RH reconstruction. In this case, it is necessary to separate or at least concentrate the phytoliths. We developed a filtration protocol for this purpose. We tested the protocol on 31 lacustrine and peat sedimentary samples from West and Central Africa, and these are the main results:

• Phytolith concentrations increased in 23 samples, primarily due to the removal of long pennate diatom frustules and sponge spicules. Six samples showed no significant change in phytolith concentration, while two samples showed a decrease.
• Twenty-nine samples achieved final phytolith proportions exceeding 40 % and a sensitivity analysis based on an isotope mass balance equation confirmed that these samples are suitable for ^17^O-excess measurements to reconstruct RH.

## Graphical abstract

**Figure:**
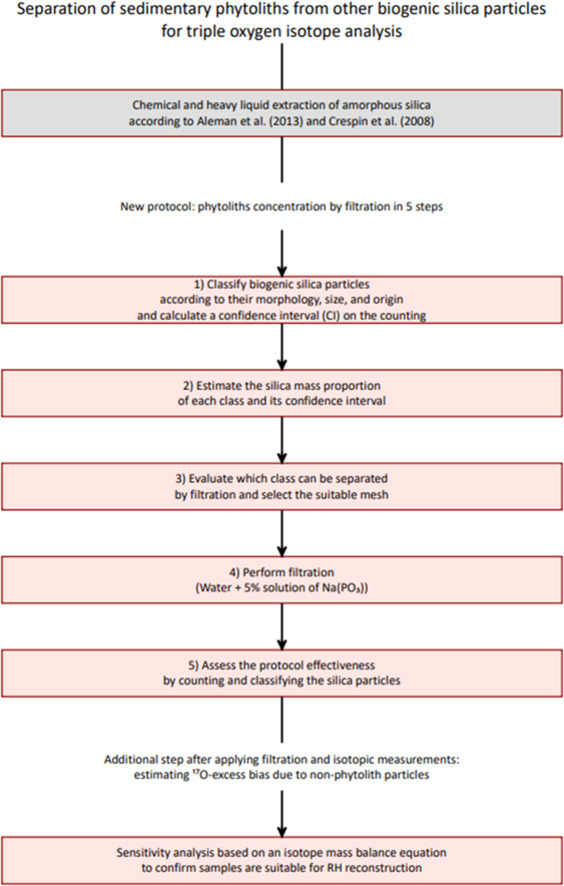

Specifications table

**Table:** 

Subject area:	Environmental Sciences
More specific subject area:	Silica extraction and separation
Name of your method:	Phytoliths concentration by separating from others silica particles by filtration
Name and reference of original method:	Kelly, 1990; [1,2]
Resource availability:	Appendix 1 for supplementary data

## Background

Phytoliths are micrometric biogenic particles of hydrated amorphous silica (SiO_2_, nH_2_O) resulting from the absorption by plant roots of silica in colloidal or dissolved form and which polymerize in all plant organs. Phytoliths can imprint the morphology of the plant cell and/or intercellular area in which they form, making it possible to identify the type of plant from which they originate. For instance, it is possible to identify grasses at the sub-family level and phytoliths derived from woody tropical dicotyledons [[3], [4], [5]]. The morphological phytolith assemblages of tropical forests are thus distinct from those produced by savannas [6], and fossil phytolith assemblages can therefore be used to reconstruct past vegetation changes, such as shifts between forests and savannas [7,8].

Additionally, recent calibrations have shown that the triple oxygen isotope composition of phytoliths is a quantitative proxy for atmospheric relative humidity (RH) [[9], [10], [11]]. Phytoliths precipitate in isotope equilibrium with plant water, and RH drives the extent of leaf water isotope fractionation during transpiration [12,13], which is then reflected in the triple oxygen isotope composition of phytoliths expressed by the ^17^O-excess (Eq. (1)):

(1) ^17^O-excess=δ’^17^O – 0.528 * δ’^18^O

With δ’ = $1000*\left(\frac{\delta }{1000}+1\right)$

A quantitative relationship linking ^17^O-excess and RH has been calibrated in growth chamber and natural environments [9,10]. While the calibration was performed using phytoliths extracted from plants and soils, reconstructing past RH requires the analysis of fossil phytoliths extracted from natural archives such as lacustrine or peat sedimentary cores. However, the chemical and heavy liquid separation methods used for phytolith extraction from sediments [1,2] extracts all amorphous silica particles including diatom frustules, sponge spicules, or volcanic ashes. These particles have distinct oxygen isotope signatures compared to phytoliths; for instance, sponge spicules and diatom frustules precipitate in isotope equilibrium with lake or peat water [2,14,15], which are less evaporated than plant water. Since each silica type has a different isotope signature, the isotope composition of a bulk amorphous silica sample represents an averaged signal, complicating its interpretation in terms of RH reconstruction.

We propose here a protocol for concentrating phytoliths, a prerequisite for reconstructing past RH using their ^17^O-excess signature. To achieve this, a filtration step is added to the chemical and densimetric extraction of amorphous silica. This step aims to remove large non-phytolith particles, such as diatom frustules and sponge spicules, thereby increasing the relative concentration of smaller phytoliths in the samples. The detailed filtration protocol is described below.

## Method details

### Material

Thirty-one samples of peat and lacustrine sediments collected in West and Central Africa were processed (Table 1). Three are peat samples collected in Republic of Congo (ROC) and Cameroon [16]. Twenty one are sediment samples from Lake Ngofouo in ROC [7]. The others are constituted of the top centimetres from lake sediment cores collected in Cameroon [17] and ROC.

**Table 1:** Sample location and description and mesh used for filtration. L.*s* = Lake sediment.

Sample_ID	Country	Latitude (°)	Longitude (°)	Site	Type	Sediment depth (cm)	Mesh sieve used (µm)
RC2234	ROC	−0.8113	16.7099	Passi Ya Kanda	Peat	0–15	40
RC2229	ROC	−0.8104	16.7114	Passi Ya Kanda	Peat	0–15	40
NGBA19	Cameroon	7.13	13.7	Ngaoundaba	Peat	12.5–15	40
C1	ROC	−3.86976	14.9489	Ngofouo	L.s	841–842	40
C4	ROC	−3.86976	14.9489	Ngofouo	L.s	786–787	40
C5	ROC	−3.86976	14.9489	Ngofouo	L.s	780–781	40
C6	ROC	−3.86976	14.9489	Ngofouo	L.s	776–777	40
C7	ROC	−3.86976	14.9489	Ngofouo	L.s	770–771	40
C8	ROC	−3.86976	14.9489	Ngofouo	L.s	765–766	40
C9	ROC	−3.86976	14.9489	Ngofouo	L.s	750–751	40
C10	ROC	−3.86976	14.9489	Ngofouo	L.s	730–731	40
C11	ROC	−3.86976	14.9489	Ngofouo	L.s	710–711	40
C12	ROC	−3.86976	14.9489	Ngofouo	L.s	690–691	40
C14	ROC	−3.86976	14.9489	Ngofouo	L.s	650–651	40
C15	ROC	−3.86976	14.9489	Ngofouo	L.s	630–631	40
C19	ROC	−3.86976	14.9489	Ngofouo	L.s	778–779	40
C20	ROC	−3.86976	14.9489	Ngofouo	L.s	759–760	40
C21	ROC	−3.86976	14.9489	Ngofouo	L.s	735–736	40
C22	ROC	−3.86976	14.9489	Ngofouo	L.s	725–726	40
C23	ROC	−3.86976	14.9489	Ngofouo	L.s	681–682	40
C24	ROC	−3.86976	14.9489	Ngofouo	L.s	663–664	40
C25	ROC	−3.86976	14.9489	Ngofouo	L.s	658–659	20
C26	ROC	−3.86976	14.9489	Ngofouo	L.s	614–615	20
C27	ROC	−3.86976	14.9489	Ngofouo	L.s	580–582	40
MLO	ROC	−3.362	14.507	Moulouomo	L.s	0–6	20
MAMG	Cameroon	8.3909	13.7089	Mamguieva	L.s	1–4	40
BARO	Cameroon	4.6544	9.4086	Barombi Mbo	L.s	1–4	20
ASSO	Cameroon	6.6245	12.9816	Assom	L.s	1–4	40
TABE	Cameroon	7.1307	13.694	Tabéré	L.s	1–4	40
TIZO	Cameroon	7.2533	13.5769	Tizong	L.s	1–4	40
NDO23–1	Cameroon	5.6697	10.536243	Ndoumkain	L.s	0.15–1.15	20

### Chemical and heavy liquid extraction of amorphous silica

**Fig. 1:**
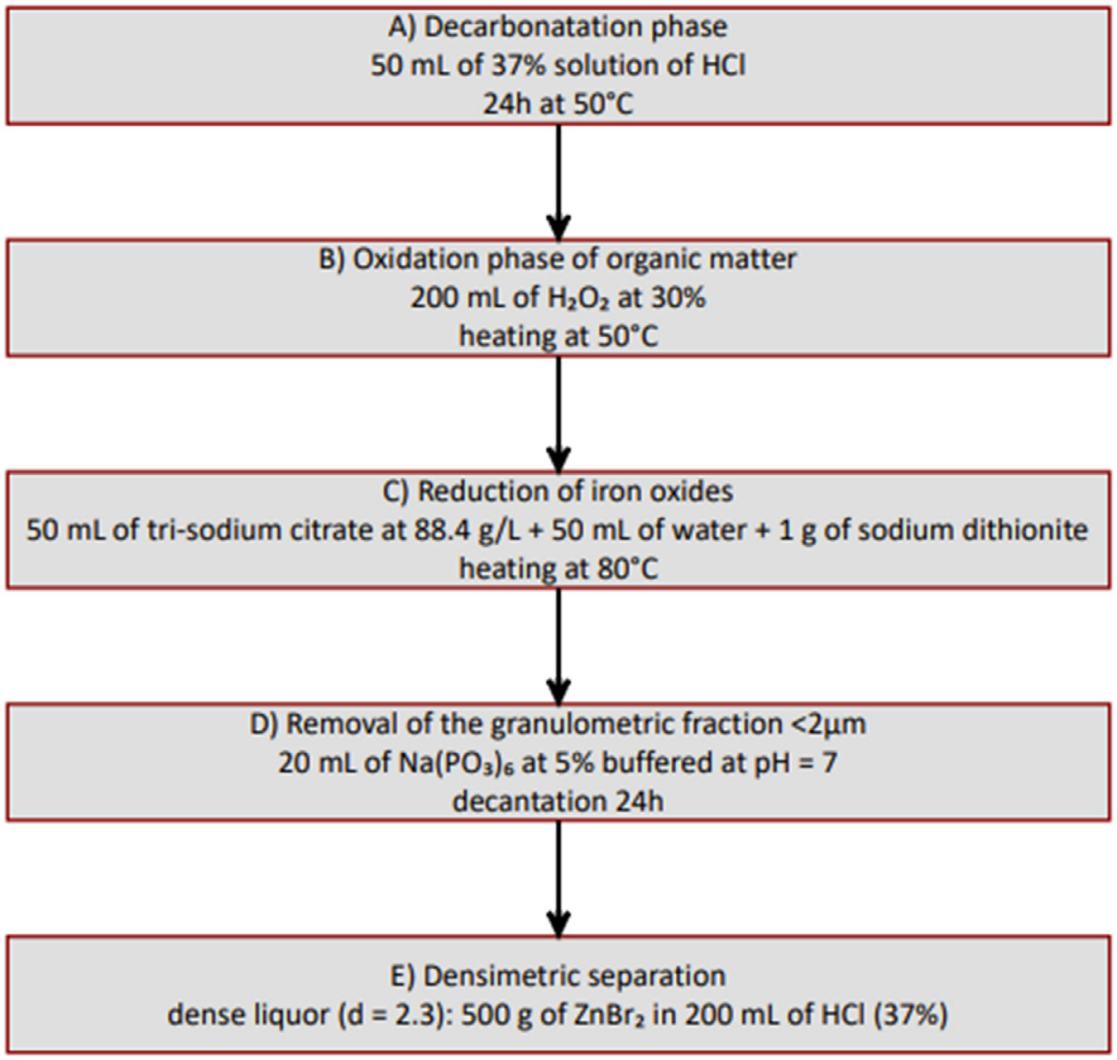
Steps for extracting biogenic silica from lacustrine or peat sediments for oxygen isotope analysis. The protocol applies to 1 to 3 g of dry initial material.

Bulk amorphous silica was extracted from the sediments using the protocol detailed in Crespin et al. [2] and Aleman et al. [1] and summarized in Fig. 1 (from A to E steps). For oxygen isotope analysis, maximum temperature values during heating phases (50 °C and 80 °C) should be respected to avoid possible dissolution that would modify the initial oxygen isotope composition.

### New 5 steps protocol for phytolith concentration using filtration

**Fig. 2:**
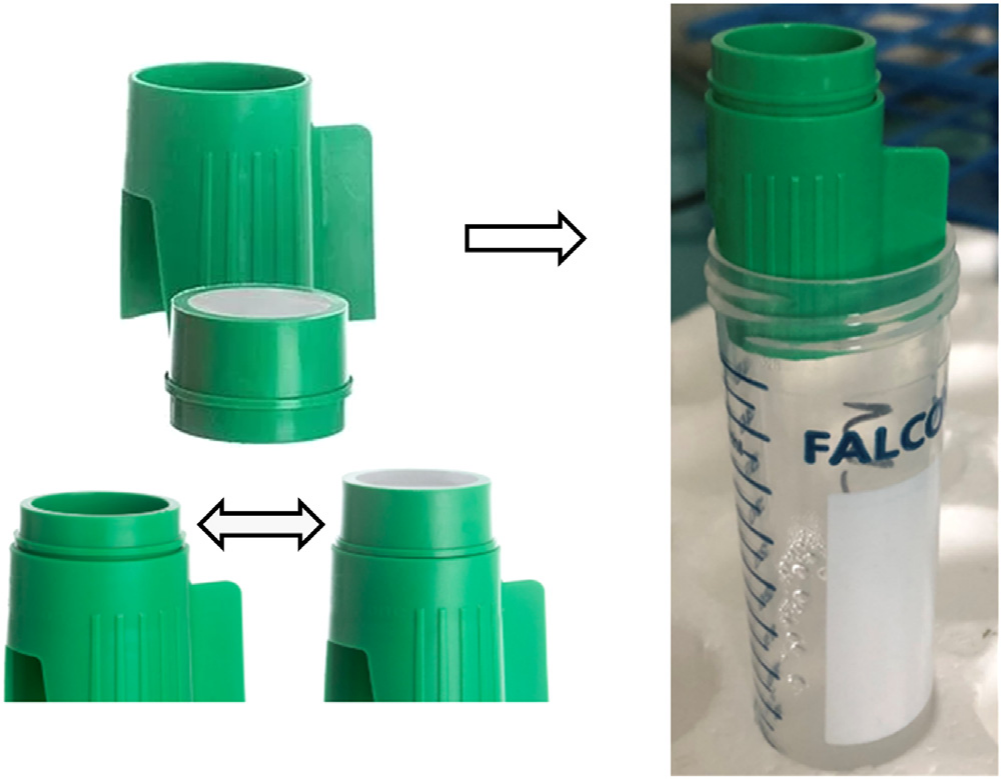
EASYstrainer© Small cellular sieve, 40 µm mesh size with invertible upper part.

Filtration was chosen as the separation method because amorphous silica particles, including phytoliths, diatom frustules, and sponge spicules, have similar densities (ranging from 2.1 to 2.3 g.cm^3^), making densimetric separation ineffective. After extracting the amorphous silica, samples were examined under a microscope to identify the type and biological origin of the particles, and to estimate their size in order to compute the mass proportion of each particle types. If the sizes of phytoliths, diatom frustules and sponge spicules differed sufficiently, filtration was applied to separate them. We used 20 and 40 µm cellular sieves (EASYstrainer©, see Fig. 2) to remove sponge spicules and diatom frustules which were longer than 20 or 40 µm.

**Fig. 3:**
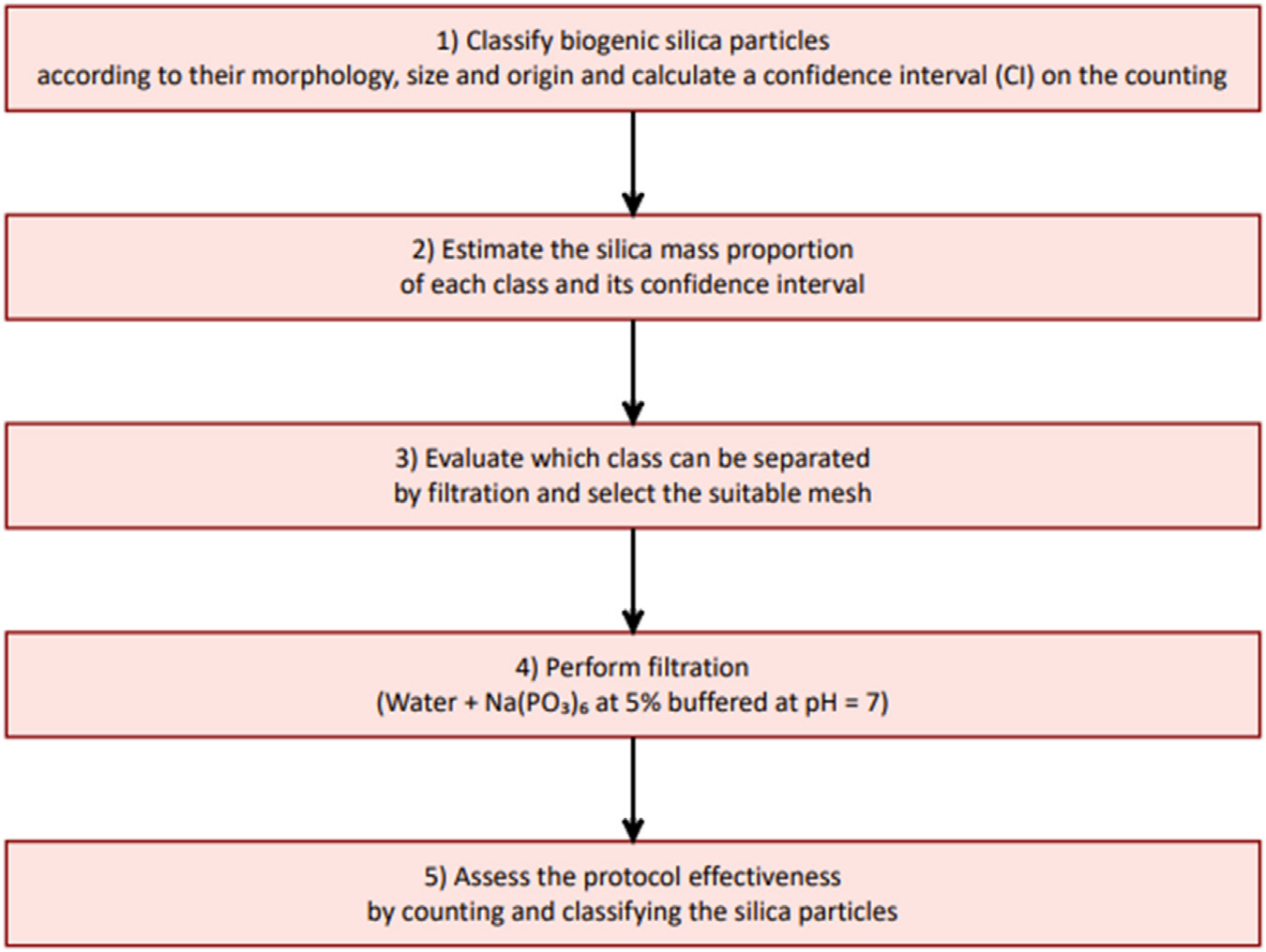
Five new steps for concentration of phytoliths by filtration from bulk amorphous silica samples.

The choice of the mesh size depends on the sample composition, and should be adapted as needed when applying this protocol in other contexts. In our study, the sieve sizes were selected to best suit our specific assemblages; but other sieve models may also be suitable depending on the specific requirements of different studies. To evaluate the effect of filtration on phytoliths, we compared the quantity (in mass proportion) and types of phytoliths, diatom frustules and sponge spicules before and after filtration, allowing us to identify which particles were removed (details in Appendix 1). The complete five-step sieving protocol is detailed below and summarized in Fig. 3.

**Step 1)** The amorphous silica particles counted under a microscope are classified based on their biological origin, morphology and size. A confidence interval (CI, Eq. (2)) around the proportion of each class with a particle count ≥ 10 can be estimated [18]:
  (2) $$\text{CI}\left(p\right)=\frac{n_{p}}{n}\;\pm \;Z_{1-\alpha /2}\times \;\sqrt{\frac{\frac{n_{p}}{n}\;\times \;\left(1-\frac{n_{p}}{n}\right)}{n}}$$

With *p* the *true* proportion of one class of silica particles, *n_p_* the number of particles counted for this class, *n* the total number of silica particles counted and $Z_{1-\alpha /2}$ the 1 − α / 2 percentile of a standard normal distribution (1.96 for a 95 % CI). For classes with a particle count < 10, an uncertainty of ± 2 is applied to the counting, because the formula above is not adapted to small counts.

For some samples, the counting was repeated 2 to 3 times, and the proportion of each class consistently fell within the CI calculated using Eq. (2).

**Step 2)** The silica mass proportion is estimated using the formula specific to each morphological class (Table 2). The volume proportion is then multiplied by the density (2.1 for diatom and sponge spicules, 2.3 for phytoliths) to obtain the mass of silica. Assuming that the error in mass calculation remains constant, the CI associated with the counting can be applied to the silica mass proportion estimation.

**Table 2:** Volume calculation for parallelepipedal, cylindrical and spherical particles, which phytoliths, diatom frustules and sponge spicules were assigned to (see Fig. 4 for visualizing the different classes). With n_p_ the number of particles counted for a class (with an associated CI), L the average length of the particles, w the average width, t the average thickness, r the average radius, and p a perforation factor which correspond to perforations in the ornamentation of diatom frustules (e.g. Fig. 4.C.b). The volume of each particle type is then multiplied by its density to calculate the mass of silica.

Morphology	Silica volume	Corresponding types from Fig. 4
parallelepiped	n_p_ x [L x w x t x *p*]	A-(c-l) ; C-(c-h)
cylinder	n_p_ x [πx L x (r² - (r-t)²) x *p*], with a hollow n_p_ x [πx L x r² x *p*], otherwise	B-(a-d); C-(a-b)
sphere	n_p_ x [$\frac{4}{3}$ πx*r^3^]	A-(a-b)

**Step 3)** A mesh size is selected to sieve the longest non-phytolith particles, while minimizing the loss of phytoliths. In our samples, the length of diatom frustules and sponge spicules was often greater than 20 or 40 µm, whereas most phytoliths were shorter; therefore, we used 20 or 40 µm cellular sieves. To select the optimal mesh size between the two available options, we simulated filtration outcomes by numerically excluding all particles larger and/or longer than the selected mesh size from the initial counts (assuming 100 % removal of these particles) and computed a theoretical filtration result (see Appendix 1). The sieves were mounted on centrifuge tubes (Fig. 2). They are washable and reusable across multiple samples.
**Step 4)** The samples are processed as follows:

a. Add a deflocculant (e.g., a sodium metaphosphate solution (Na(PO_3_)_6_) buffered at pH = 7) to achieve a good dispersion of the particles.
b. Filter the sample through the selected cellular sieve. While filtering, stir the solution manually using a Pasteur pipette to facilitate the passage of the small particles through the mesh.
c. Centrifuge the filtrate.
d. Repeat the filtration process with the same mesh until the filtrate becomes transparent.

**Step 5)** The mass proportion of phytoliths in the filtrate is compared to that in the initial assemblage (referred to as the “Initial” fraction below) to assess which particles were removed by filtration. Filtration is considered effective if the phytolith mass proportion in the filtrate exceeds the upper bond of the confidence interval (CI) of the initial mass phytolith proportion, indicating a concentration of phytoliths relative to other silica particles. In cases where the phytolith proportion decreases after filtration, we recommend performing a second one using a different mesh size to improve the removal of non-phytolith particles while trying to minimize phytolith loss.

### Estimating ^17^O-excess bias due to non-phytolith particles

Phytoliths precipitate in equilibrium with plant waters, which show the largest variations in triple oxygen isotope composition among surface waters [10,19,20]. These variations result from the leaf being a small water reservoir with limited contribution of unevaporated water circulating in its veins, making leaf water highly responsive to rapid RH changes [10,[19], [20]–21]. In contrast, sponge spicules and diatom frustules precipitate from lake or peat waters [2,14,15], where the proportion of evaporated water is much lower. The ^17^O-excess of bulk amorphous silica samples containing both phytoliths and sponge spicules and/or diatoms reflects the influence of at least two water reservoirs with distinct isotope compositions.

We performed a sensitivity analysis to quantify the deviation of the ^17^O-excess of bulk amorphous silica samples from the ^17^O-excess of phytoliths (used for RH reconstruction) due to non-phytolith particles. The ^17^O-excess of a bulk sample can be expressed using an isotope mass balance equation (Eq. (3)):

(3) $${}^{17}O-\text{exces}s_{\text{sample}}=x_{\text{phyto}}\times {}^{17}O-\text{exces}s_{\text{phyto}}+\left(1-x_{\text{phyto}}\right)\;\times {}^{17}O-\text{exces}s_{\text{diat}\_\text{sponge}}$$

where x_phyto_ represents the mass proportion of phytoliths in the sample; ^17^O-excess_sample_ the measured triple isotope composition of a bulk sample; ^17^O-excess_phyto_ the ^17^O-excess of phytoliths in the sample; and ^17^O-excess_diat_sponge_ the ^17^O-excess of non-phytolith particles (e.g., diatom frustules and sponge spicules) in the sample.

The sensitivity analysis was conducted by simulating ^17^O-excess values for a sample (^17^O-excess_sample_) computed using Eq. (3) and with values of x_phyto_ ranging from 0.05 to 0.95 and ^17^O-excess_phyto_ values ranging from −150 to −300 per meg, in agreement with grass leaf phytoliths grown under RH ranging from 40 to 80 % [9,10]. Since measured ^17^O-excess values of sponge spicules and diatom frustules are unavailable, we estimated a plausible range of δ^18^O and ^17^O-excess (^17^O-excess_diat_sponge_) based on the following assumptions: 1) there is isotopic equilibrium between lake or peat water and sponge spicules or diatom frustules; 2) their forming water is little evaporated, such that their triple oxygen isotope composition close to that of precipitation. Using silica-water equilibrium fractionation factors from Dodd & Sharp [22] and Sharp et al. [23], along with the triple oxygen isotope composition of precipitation recently published for sites in Central Africa [24], and assuming a temperature of 25 °C, we calculated ^17^O-excess_diat_sponge_ values ranging between −160 and −195 per meg. These values were used in the simulation of ^17^O-excess_sample_ in Eq. (3).

All numerical simulations and graphs were performed using R software (R version 4.2.2, R Core Team, [25]) with *ggplot2, dplyr, viridisLite* packages.

## Method validation

### Application to lacustrine and peat sediment samples

**Fig. 4:**
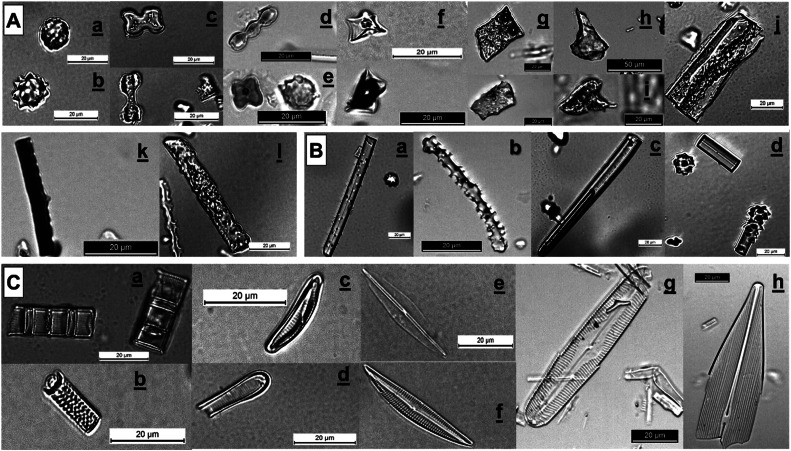
Morphological types of phytoliths according to the International Code for Phytolith Nomenclature (ICPN) 2.0 [26], diatom frustules according to Round et al. [27] for the differentiation between pennate and centric diatom frustules, and sponge spicules found in the samples. *A* = phytolith types: (a-b, spherical) spheroid, (c-f, parallelepipedal) Grass silica short-cell phytoliths (GSSCP), (g, parallelepipedal) blocky, (h, parallelepipedal) bulliform, (i, parallelepipedal) acute bulbosus, (j, parallelepipedal) tabular, (k-l, parallelepipedal) elongate. *B* = sponge spicule: (a-c, cylindrical) long sponge spicule (*L* > 40–20 µm), (d, cylindrical) small sponge spicules (*L* < 40–20 µm). *C* = diatom frustules types: (a cylindrical) centric colonies, (b, cylindrical) centric, (c-d, parallelepipedal) small pennate (*L* < 40–20 µm), (e-h, parallelepipedal) long pennate (*L* > 40–20 µm).

Phytoliths, diatom frustules and sponge spicules were categorized into parallelepipedal, cylindrical and spherical types of particles larger or smaller than 20–40 µm, resulting in a total of 11 classes (Fig. 4 and Appendix 1).

**Fig. 5:**
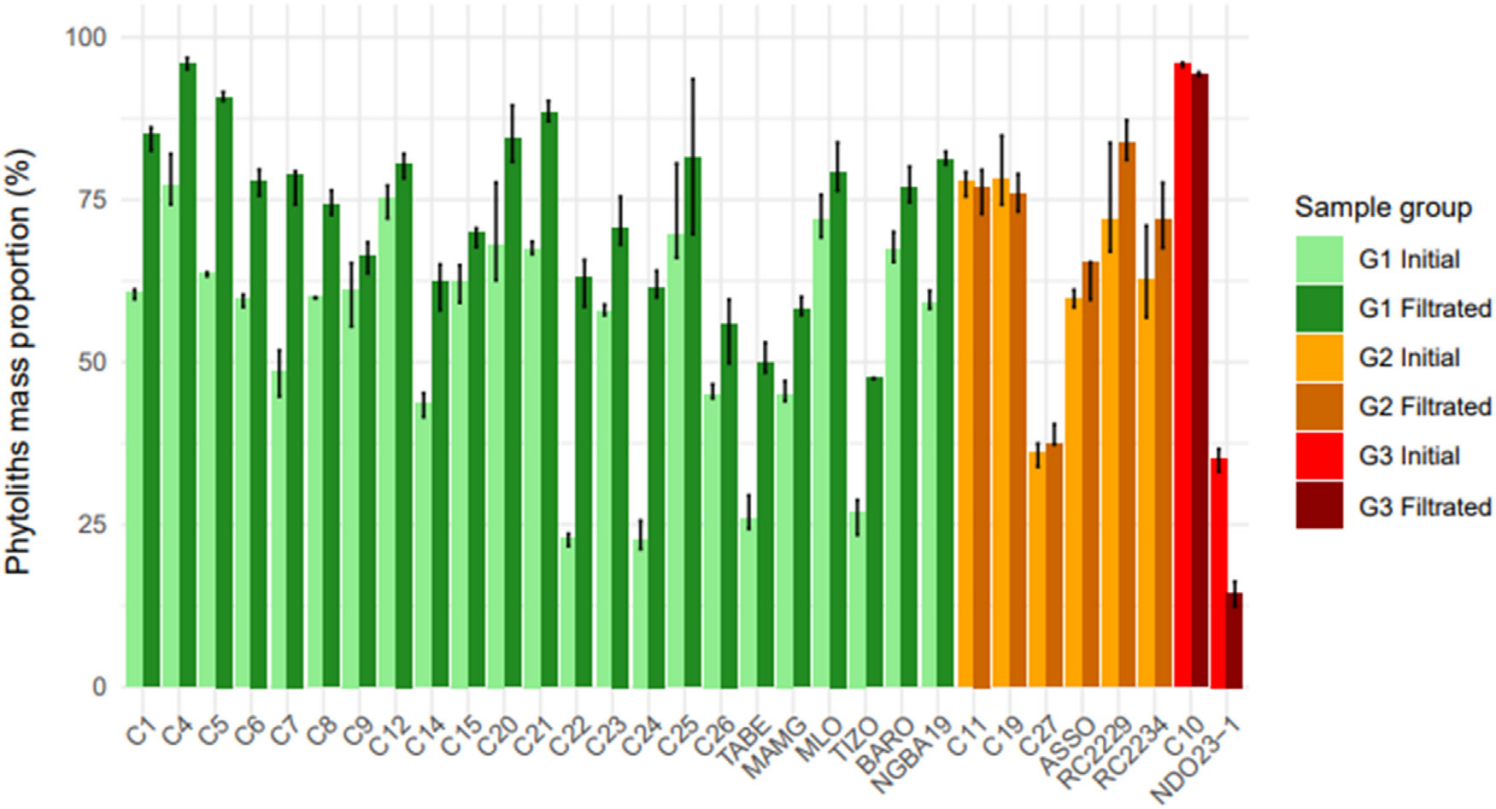
Phytoliths mass proportion before (Initial) and after (Filtrated) filtration for the 31 samples. Three groups can be identified based on the observed changes in phytoliths mass proportions: significant increase (G1), no significant change (G2), and significant decrease (G3).

Phytoliths represented between 11 % and 87 % of the initial amorphous silica particles counted. The mass of each class was calculated (Table 2), with initial mass proportions for phytoliths ranging between 22 ± 2 and 95 ± 0.3 % (Fig. 5 and Appendix 1).

Of the 31 samples tested (Fig. 5), 23 showed a significant increase in the mass proportion of phytoliths after filtration, with an average increase of 18 % ± 4 (group 1 = G1), 6 showed no significant changes in the mass proportion of phytoliths (group 2 = G2), while 2 samples showed a significant decrease (group 3 = G3).

- Group 1: increase in phytoliths mass proportion

**Fig. 6:**
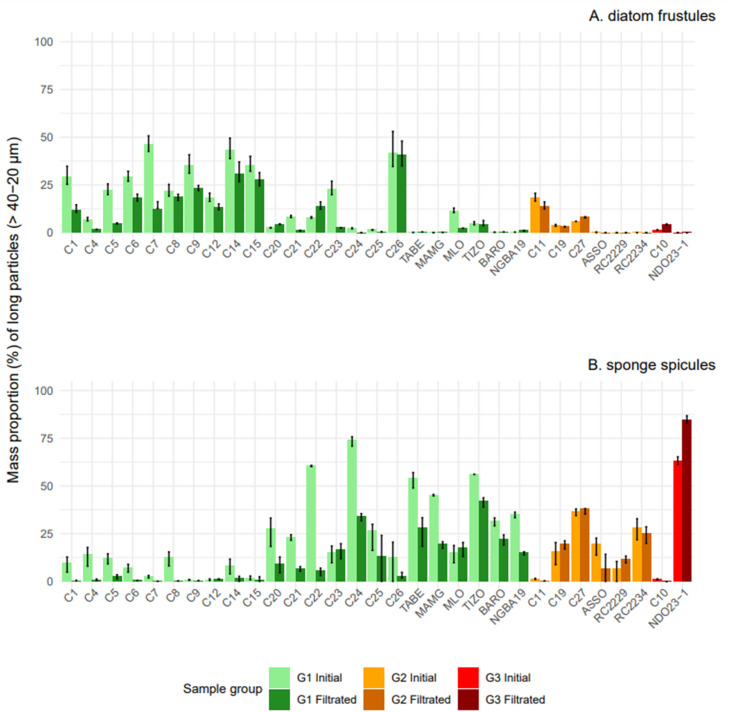
Long pennate diatom frustules (A.) and sponges spicules (B.) mass proportion before (Initial) and after (Filtrated) filtration for the three groups identified above. Particles are classified as 'long' when their average length exceeds the mesh size (20 µm or 40 µm).

Across the 23 samples of this group (Fig. 5), filtration at 40 µm or 20 µm successfully concentrated phytoliths by eliminating long pennate diatom frustules and/or sponge spicules (Fig. 6, Appendix 1). Three distinct patterns across samples were observed: (1) a decrease in both pennate diatom frustules and sponge spicules (C1, C4, C5, C6, C7, C14, C21); (2) a decrease primarily in pennate diatom frustules (C9, C12, C15, C23, MLO); and (3) a decrease mainly in sponge spicules (C8, C20, C22, C24, C25, C26, TABE, MAMG, TIZO, BARO, NGBA19). This means that filtration was particularly effective when the initial assemblages of non-phytolith particles were dominated by long pennate diatoms and/or long sponge spicules, which were much longer than the phytoliths. The large specific surface area of pennate diatom frustules increased their likelihood of being retained by the sieve, while the significant length of sponge spicules (> 100 µm) also facilitated their retention.

- Goup 2: no change in phytoliths mass proportion

Filtration did not significantly modify the phytoliths mass proportion in six samples (16 %), as their proportions remained within the CI of the initial values (Fig. 5, Appendix 1). This lack of change is attributed to the absence of significant shifts in the size distribution of sponge spicules and diatom frustules (e.g. C19 and C27). In other cases, while long pennate diatom frustules and sponge spicules were removed, this effect was offset by the removal of some phytolith types. For instance, some spheroid phytoliths in ASSO, elongate phytoliths in C11 and GSSCP phytoliths in C19 were removed. In these cases, the remaining assemblages were still dominated by diatom frustules and/or sponge spicules smaller than the mesh size (< 40–20 µm), which could not be effectively separated by filtration (Appendix 1). Finally, RC2229 and RC2234 showed an average increase in phytoliths mass proportion of 10.4 % ± 7.3 but the wide CI in their initial phytolith mass proportions make this increase non-significant.

- Group 3: decrease in phytoliths mass proportion

In two cases, filtration led to a decrease in the mass proportion of phytoliths (Fig. 5), mainly due to the loss of certain types of large phytoliths (spheroid, blocky and bulliform phytoliths), which did not compensate for the removal of non-phytolith particles (Appendix 1, Fig. 6). Additionally, long sponge spicules with small diameters passed through the sieve, further contributing to their increased mass proportion. This highlights a limitation of the method: while effective for concentrating smaller phytoliths, it may lead to selective loss of larger phytolith morphotypes.

For NDO23–1 (group 3) and BARO (group 1), we applied a second filtration with a smaller mesh size (20 µm) to remove smaller sponge spicules and diatom frustules. This second filtration was effective for BARO (Appendix 1, Fig. 5), for which the first filtration at 40 µm had resulted in a decrease in phytolith proportion. For NDO23–1, however, the second filtration at 20 µm was ineffective due to the presence of many sponge spicules small enough to pass through the sieve (Appendix 1). In this case, removing long sponge spicules did not sufficiently compensate for the large number of smaller ones, nor for the loss of large phytoliths, even after adjusting the mesh size.

Among the 31 samples, 27 had a final phytoliths mass proportion ≥ 50 %, including 24 samples were ≥ 60 %, 16 samples ≥ 75 %, and 4 reaching or exceeding 87.5 %.

### ^17^O-excess bias due to non-phytolith particles

**Fig. 7:**
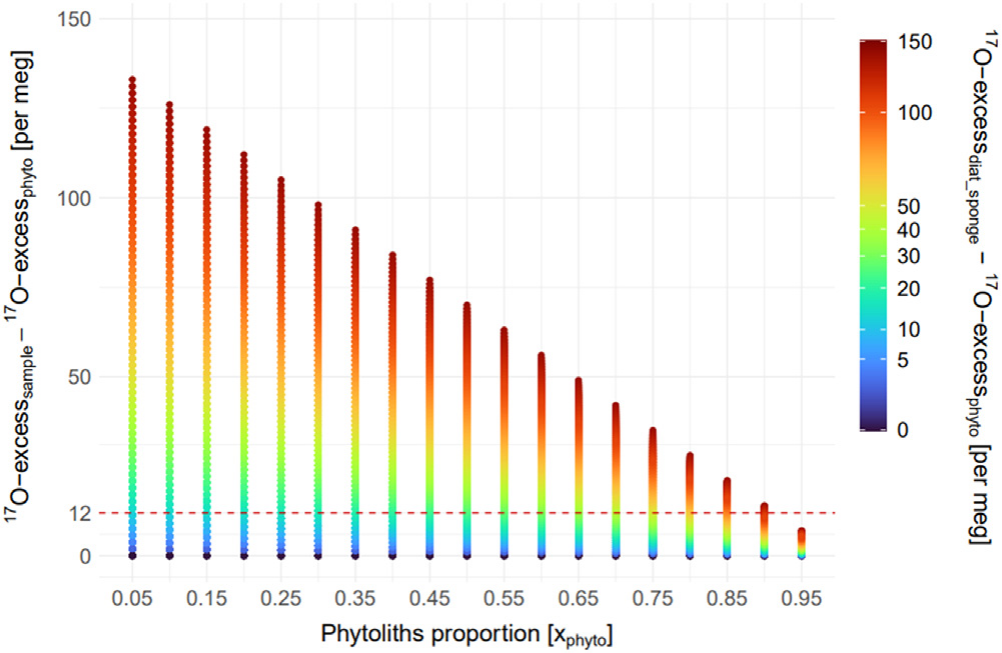
Sensitivity analysis showing the bias in ^17^O-excess measurements, assessed using the difference between ^17^O-excess_xample_ and ^17^O-excess_phyto_ (Y-axis), as a function of phytoliths proportion (x_phyto_, X-axis) and the difference between ^17^O-excess_diat_sponge_ and ^17^O-excess_phyto_ (color-code). The red dashed line at 12 per meg indicates the analytical precision threshold [10] above which measurements are less reliable for RH reconstructions.

The sensitivity analysis assesses the extent to which ^17^O-excess_sample_ deviates from ^17^O-excess_phyto_ based on phytoliths proportion and the isotopic composition of phytolith and non-phytolith particles derived from the literature (Fig. 7). The results showed values ranging from 133 to 41.8 per meg for the difference between ^17^O-excess_sample_ and ^17^O-excess_phyto_, and from 140 to 44 per meg of difference between ^17^O-excess_diat-sponge_​ and ^17^O-excess_phyto_. The presented differences are absolute values to facilitate the readability of the graph, which provides a framework for aiding the interpretation of measured data in terms of reliability for RH reconstructions.

As phytoliths proportion in a sample increases, the deviation between ¹⁷O-excess_sample_ and ¹⁷O-excess_phyto_ decreases, thereby reducing the bias introduced by other silica particles. At the same time, the influence of the differences in isotopic composition between phytoliths and non-phytoliths particles (¹⁷O-excess_diat_sponge_ - ¹⁷O-excess_phyto_) on this deviation also decreases. The results highlight ranges for ensuring reliable RH reconstructions.

For low phytoliths proportions (between 5 % and 35 %), when the difference between ¹⁷O-excess_diat_sponge_ and ¹⁷O-excess_phyto_ exceeds 10 per meg (for x_phyto_ around 5 %) to 15 per meg (for x_phyto_ around 35 %), the difference between ¹⁷O-excess_sample_ and ¹⁷O-excess_phyto_ - therefore the bias - surpasses the analytical precision threshold of 12 per meg, making RH estimates unreliable. Indeed, even small differences in isotopic composition between phytoliths and non-phytoliths particles significantly affect the measurement.

For intermediate phytoliths proportions (between 40 % and 70 %), the bias exceeds the analytical precision threshold when the difference between ¹⁷O-excess_diat_sponge_ and ¹⁷O-excess_phyto_ ranges from 20 per meg (for x_phyto_ around 40 %) to 40 per meg (for x_phyto_ around 70 %). These samples generally provide reliable isotope measurements for RH reconstructions; however, caution is needed when interpreting the results.

For high phytoliths proportions (between 75 % and 95 %), the bias stays below the analytical precision unless the difference between ¹⁷O-excess_diat_sponge_ and ¹⁷O-excess_phyto_ exceeds 50 per meg.

For very high phytoliths proportions (> 95 %), the bias always remains below the analytical precision, regardless of the isotope difference between phytoliths and non-phytoliths particles.

## Limitations

In 75 % of the samples, phytolith mass proportion increased, confirming that our filtration protocol is generally effective in concentrating phytoliths extracted from lacustrine and peat samples. Filtration using 20 µm or 40 µm mesh sizes was most effective when the initial biogenic silica assemblage was dominated by long particles, such as long pennate diatom frustules or sponge spicules, which differed significantly in size from phytoliths. While diatom frustules with very large specific surface area and long sponge spicules (> 100 µm) were particularly well retained by the mesh sieve, some non-phytolith particles still persisted in the filtrated samples.

Our protocol still has limitations: while it is effective for concentrating smaller phytoliths, it may lead to the selective loss of larger or longer phytolith morphotypes. In particular, spheroid or blocky types, may be removed if they exceed the mesh size (e.g., 40 µm). In some cases, the filtration remained effective because the removal of non-phytolith particles was enough to compensate for phytoliths loss, but this was not the case for group 3. Adjusting the filter mesh size can be a potential solution, but it would not fully resolve the issue. The large size variability among phytoliths and the different shapes of particles in natural assemblages make it difficult to selectively retain all phytoliths while removing long, fine non-phytolith particles such as diatom frustules and sponge spicules. Even with a mesh designed with different length and width dimensions, blocky or spheroid phytoliths are more likely to be removed because their width could exceed the mesh opening. Thus, while mesh size is a critical factor, it cannot completely eliminate the trade-off between concentrating phytoliths and the selective loss of some larger phytolith morphotypes. Moreover, as filtration may alter the proportional composition of phytolith assemblages, analyses intended for vegetation reconstruction should be performed on the initial sample before filtration.

Assessing the bias introduced by residual non-phytolith amorphous silica particles is particularly important for RH reconstructions. Our sensitivity analysis indicates that samples with a final phytolith proportion above 75 % can reliably reflect the triple oxygen isotope signature of phytoliths, provided that the difference in triple oxygen isotope signatures between phytoliths and non-phytoliths remains below 50 per meg. Under these conditions, it is unlikely that the analytical precision threshold will be exceeded, despite the presence of non-phytolith particles. Among the 31 samples tested, 16 had a final phytolith proportion above 75 % and can therefore be considered reliable for RH reconstructions. Samples with a final phytolith proportion between 40 % and 75 %, representing 13 of our samples, should be interpreted with caution unless the difference in triple oxygen isotope signatures between phytoliths and non-phytoliths is <20 to 40 per meg. Samples with a phytolith proportion below 40 %, two samples in our case, should be considered as mixtures and their low phytolith concentration may contribute to unreliable.

Finally, when the initial phytolith proportion is already high (> 75 %), further concentration of phytoliths is still recommended to improve the accuracy and reliability of the ^17^O-excess values and improve RH reconstructions. This highlights the importance of optimizing phytolith concentration before triple oxygen isotopes measurements.

To conclude, this study presents the first protocol to specifically concentrate phytoliths from other silica particles, such as diatoms and sponge spicules, through filtration. This filtration step is added to the existing chemical and density phytolith extraction method, and ensure concentrating phytoliths in sediment samples from lakes or peatlands. The mesh size used in the filtration should be adapted to the initial assemblage of each sample, taking into account the morphology and size of the different particles. Following filtration and the measurement of the triple oxygen isotope composition, the sensitivity analysis developed here provides a tool to assess the reliability of measured isotopic values for RH reconstructions, based on the phytolith proportions obtained after filtration. This final additional step helps evaluate the potential biases introduced by non-phytolith particles and should be adjusted to the specific characteristics of each user's samples.

## Ethics statements

Not applicable

## CRediT authorship contribution statement

**Charlotte Mention:** Investigation, Methodology, Formal analysis, Visualization, Writing – original draft. **Julie C. Aleman:** Conceptualization, Methodology, Formal analysis, Supervision, Project administration, Funding acquisition, Writing – review & editing. **Jean-Charles Mazur:** Methodology, Resources, Writing – review & editing. **Yannick Garcin:** Resources, Writing – review & editing. **Christelle Hély:** Supervision, Writing – review & editing. **Anne Alexandre:** Conceptualization, Methodology, Writing – review & editing, Funding acquisition.

## Declaration of competing interest

The authors declare that they have no known competing financial interests or personal relationships that could have appeared to influence the work reported in this paper.

## Data Availability

Data will be made available on request.

## Appendix Supplementary materials

Image, application 1
